# Supplementary material for: Pyruvate Dehydrogenase Kinase 1 inhibition mediated oxidative phosphorylation enhancement in cartilage promotes osteoarthritis progression
Source: BMC Musculoskelet Disord. 2023 Jul 20;24:597. doi: 10.1186/s12891-023-06585-6 (PMC10357736; doi:10.1186/s12891-023-06585-6)
Supplement: Supplementary file 1 — Additional file 1. [file 12891_2023_6585_MOESM1_ESM.zip › BP.Gsea.1653623667859/GOBP_GENE_SILENCING_BY_RNA.html]

Details for gene set GOBP\_GENE\_SILENCING\_BY\_RNA[GSEA]

|  || Dataset | 55457\_collapsed\_to\_symbols.cls.cls #OA\_versus\_normal.cls.cls #OA\_versus\_normal\_repos |
| Phenotype | cls.cls#OA\_versus\_normal\_repos |
| Upregulated in class | normal |
| GeneSet | GOBP\_GENE\_SILENCING\_BY\_RNA |
| Enrichment Score (ES) | -0.403327 |
| Normalized Enrichment Score (NES) | -1.7304884 |
| Nominal p-value | 0.0 |
| FDR q-value | 0.12279725 |
| FWER p-Value | 1.0 |
Table: GSEA Results Summary

  

Fig 1: Enrichment plot: GOBP\_GENE\_SILENCING\_BY\_RNA      
 Profile of the Running ES Score & Positions of GeneSet Members on the Rank Ordered List

  

| SYMBOL | TITLE | RANK IN GENE LIST | RANK METRIC SCORE | RUNNING ES | CORE ENRICHMENT || 1 | SMAD2 | SMAD family member 2 [Source:HGNC Symbol;Acc:HGNC:6768] | 275 | 0.768 | 0.0016 | No |
| 2 | TSN | translin [Source:HGNC Symbol;Acc:HGNC:12379] | 372 | 0.717 | 0.0156 | No |
| 3 | ZMPSTE24 | zinc metallopeptidase STE24 [Source:HGNC Symbol;Acc:HGNC:12877] | 451 | 0.682 | 0.0299 | No |
| 4 | CLP1 | cleavage factor polyribonucleotide kinase subunit 1 [Source:HGNC Symbol;Acc:HGNC:16999] | 748 | 0.582 | 0.0242 | No |
| 5 | ESR1 | estrogen receptor 1 [Source:HGNC Symbol;Acc:HGNC:3467] | 984 | 0.522 | 0.0215 | No |
| 6 | PRKRA | protein activator of interferon induced protein kinase EIF2AK2 [Source:HGNC Symbol;Acc:HGNC:9438] | 1222 | 0.472 | 0.0172 | No |
| 7 | EIF4E2 | eukaryotic translation initiation factor 4E family member 2 [Source:HGNC Symbol;Acc:HGNC:3293] | 1301 | 0.458 | 0.0248 | No |
| 8 | CNOT7 | CCR4-NOT transcription complex subunit 7 [Source:HGNC Symbol;Acc:HGNC:14101] | 1676 | 0.402 | 0.0076 | No |
| 9 | DDX6 | DEAD-box helicase 6 [Source:HGNC Symbol;Acc:HGNC:2747] | 1687 | 0.401 | 0.0189 | No |
| 10 | RBM4 | RNA binding motif protein 4 [Source:HGNC Symbol;Acc:HGNC:9901] | 1970 | 0.364 | 0.0078 | No |
| 11 | DROSHA | drosha ribonuclease III [Source:HGNC Symbol;Acc:HGNC:17904] | 2054 | 0.356 | 0.0119 | No |
| 12 | TNF | tumor necrosis factor [Source:HGNC Symbol;Acc:HGNC:11892] | 2098 | 0.350 | 0.0191 | No |
| 13 | TARBP2 | TARBP2 subunit of RISC loading complex [Source:HGNC Symbol;Acc:HGNC:11569] | 2311 | 0.325 | 0.0123 | No |
| 14 | TGFB1 | transforming growth factor beta 1 [Source:HGNC Symbol;Acc:HGNC:11766] | 2490 | 0.303 | 0.0075 | No |
| 15 | NCOR1 | nuclear receptor corepressor 1 [Source:HGNC Symbol;Acc:HGNC:7672] | 2549 | 0.297 | 0.0118 | No |
| 16 | DDX4 | DEAD-box helicase 4 [Source:HGNC Symbol;Acc:HGNC:18700] | 2788 | 0.271 | 0.0014 | No |
| 17 | FXR1 | FMR1 autosomal homolog 1 [Source:HGNC Symbol;Acc:HGNC:4023] | 3012 | 0.249 | -0.0086 | No |
| 18 | LIN28A | lin-28 homolog A [Source:HGNC Symbol;Acc:HGNC:15986] | 3140 | 0.236 | -0.0114 | No |
| 19 | AGO1 | argonaute RISC component 1 [Source:HGNC Symbol;Acc:HGNC:3262] | 3430 | 0.208 | -0.0277 | No |
| 20 | PPP3CA | protein phosphatase 3 catalytic subunit alpha [Source:HGNC Symbol;Acc:HGNC:9314] | 3803 | 0.176 | -0.0515 | No |
| 21 | CNOT1 | CCR4-NOT transcription complex subunit 1 [Source:HGNC Symbol;Acc:HGNC:7877] | 3842 | 0.172 | -0.0493 | No |
| 22 | SND1 | staphylococcal nuclease and tudor domain containing 1 [Source:HGNC Symbol;Acc:HGNC:30646] | 3897 | 0.165 | -0.0486 | No |
| 23 | SMAD3 | SMAD family member 3 [Source:HGNC Symbol;Acc:HGNC:6769] | 4061 | 0.150 | -0.0568 | No |
| 24 | ZC3H7A | zinc finger CCCH-type containing 7A [Source:HGNC Symbol;Acc:HGNC:30959] | 4212 | 0.136 | -0.0645 | No |
| 25 | AGO4 | argonaute RISC component 4 [Source:HGNC Symbol;Acc:HGNC:18424] | 4346 | 0.124 | -0.0711 | No |
| 26 | MYCN | "MYCN proto-oncogene, bHLH transcription factor [Source:HGNC Symbol;Acc:HGNC:7559]" | 4554 | 0.109 | -0.0840 | No |
| 27 | TDRKH | tudor and KH domain containing [Source:HGNC Symbol;Acc:HGNC:11713] | 4565 | 0.108 | -0.0816 | No |
| 28 | CNOT8 | CCR4-NOT transcription complex subunit 8 [Source:HGNC Symbol;Acc:HGNC:9207] | 4581 | 0.106 | -0.0795 | No |
| 29 | MORC1 | MORC family CW-type zinc finger 1 [Source:HGNC Symbol;Acc:HGNC:7198] | 4734 | 0.093 | -0.0886 | No |
| 30 | NRDE2 | "NRDE-2, necessary for RNA interference, domain containing [Source:HGNC Symbol;Acc:HGNC:20186]" | 5019 | 0.071 | -0.1087 | No |
| 31 | EIF4ENIF1 | eukaryotic translation initiation factor 4E nuclear import factor 1 [Source:HGNC Symbol;Acc:HGNC:16687] | 5330 | 0.045 | -0.1315 | No |
| 32 | TP53 | tumor protein p53 [Source:HGNC Symbol;Acc:HGNC:11998] | 5511 | 0.032 | -0.1446 | No |
| 33 | CNOT2 | CCR4-NOT transcription complex subunit 2 [Source:HGNC Symbol;Acc:HGNC:7878] | 5740 | 0.012 | -0.1620 | No |
| 34 | TERT | telomerase reverse transcriptase [Source:HGNC Symbol;Acc:HGNC:11730] | 5773 | 0.010 | -0.1642 | No |
| 35 | PPARG | peroxisome proliferator activated receptor gamma [Source:HGNC Symbol;Acc:HGNC:9236] | 6006 | -0.005 | -0.1822 | No |
| 36 | EIF6 | eukaryotic translation initiation factor 6 [Source:HGNC Symbol;Acc:HGNC:6159] | 6140 | -0.015 | -0.1921 | No |
| 37 | PIWIL1 | piwi like RNA-mediated gene silencing 1 [Source:HGNC Symbol;Acc:HGNC:9007] | 6144 | -0.016 | -0.1919 | No |
| 38 | CNOT3 | CCR4-NOT transcription complex subunit 3 [Source:HGNC Symbol;Acc:HGNC:7879] | 6183 | -0.019 | -0.1943 | No |
| 39 | SNIP1 | Smad nuclear interacting protein 1 [Source:HGNC Symbol;Acc:HGNC:30587] | 6466 | -0.040 | -0.2151 | No |
| 40 | NCOR2 | nuclear receptor corepressor 2 [Source:HGNC Symbol;Acc:HGNC:7673] | 6497 | -0.042 | -0.2162 | No |
| 41 | METTL3 | "methyltransferase 3, N6-adenosine-methyltransferase complex catalytic subunit [Source:HGNC Symbol;Acc:HGNC:17563]" | 6685 | -0.055 | -0.2292 | No |
| 42 | PUM2 | pumilio RNA binding family member 2 [Source:HGNC Symbol;Acc:HGNC:14958] | 7186 | -0.095 | -0.2653 | No |
| 43 | LIMD1 | LIM domain containing 1 [Source:HGNC Symbol;Acc:HGNC:6612] | 7281 | -0.101 | -0.2697 | No |
| 44 | ELAVL1 | ELAV like RNA binding protein 1 [Source:HGNC Symbol;Acc:HGNC:3312] | 7426 | -0.113 | -0.2775 | No |
| 45 | TSNAX | translin associated factor X [Source:HGNC Symbol;Acc:HGNC:12380] | 7606 | -0.127 | -0.2877 | No |
| 46 | BMP4 | bone morphogenetic protein 4 [Source:HGNC Symbol;Acc:HGNC:1071] | 7816 | -0.142 | -0.2997 | No |
| 47 | FMR1 | FMRP translational regulator 1 [Source:HGNC Symbol;Acc:HGNC:3775] | 7944 | -0.151 | -0.3051 | No |
| 48 | TDRD1 | tudor domain containing 1 [Source:HGNC Symbol;Acc:HGNC:11712] | 7979 | -0.153 | -0.3032 | No |
| 49 | STAT3 | signal transducer and activator of transcription 3 [Source:HGNC Symbol;Acc:HGNC:11364] | 8453 | -0.190 | -0.3344 | No |
| 50 | HNRNPA2B1 | heterogeneous nuclear ribonucleoprotein A2/B1 [Source:HGNC Symbol;Acc:HGNC:5033] | 8576 | -0.200 | -0.3379 | No |
| 51 | TUT7 | terminal uridylyl transferase 7 [Source:HGNC Symbol;Acc:HGNC:25817] | 8629 | -0.203 | -0.3359 | No |
| 52 | DGCR8 | DGCR8 microprocessor complex subunit [Source:HGNC Symbol;Acc:HGNC:2847] | 8896 | -0.224 | -0.3500 | No |
| 53 | ZC3H12A | zinc finger CCCH-type containing 12A [Source:HGNC Symbol;Acc:HGNC:26259] | 9030 | -0.234 | -0.3533 | No |
| 54 | ADAR | adenosine deaminase RNA specific [Source:HGNC Symbol;Acc:HGNC:225] | 9195 | -0.247 | -0.3587 | No |
| 55 | RAN | "RAN, member RAS oncogene family [Source:HGNC Symbol;Acc:HGNC:9846]" | 9478 | -0.272 | -0.3726 | No |
| 56 | TEX15 | "testis expressed 15, meiosis and synapsis associated [Source:HGNC Symbol;Acc:HGNC:11738]" | 9710 | -0.293 | -0.3819 | No |
| 57 | HELZ | helicase with zinc finger [Source:HGNC Symbol;Acc:HGNC:16878] | 9889 | -0.310 | -0.3864 | No |
| 58 | SMAD1 | SMAD family member 1 [Source:HGNC Symbol;Acc:HGNC:6767] | 9905 | -0.312 | -0.3783 | No |
| 59 | MAP2K2 | mitogen-activated protein kinase kinase 2 [Source:HGNC Symbol;Acc:HGNC:6842] | 9932 | -0.315 | -0.3709 | No |
| 60 | PUM1 | pumilio RNA binding family member 1 [Source:HGNC Symbol;Acc:HGNC:14957] | 10145 | -0.335 | -0.3774 | No |
| 61 | MAP2K1 | mitogen-activated protein kinase kinase 1 [Source:HGNC Symbol;Acc:HGNC:6840] | 10197 | -0.340 | -0.3711 | No |
| 62 | TUT4 | terminal uridylyl transferase 4 [Source:HGNC Symbol;Acc:HGNC:28981] | 10377 | -0.359 | -0.3744 | No |
| 63 | DHX9 | DExH-box helicase 9 [Source:HGNC Symbol;Acc:HGNC:2750] | 10627 | -0.386 | -0.3822 | No |
| 64 | POU5F1 | POU class 5 homeobox 1 [Source:HGNC Symbol;Acc:HGNC:9221] | 10898 | -0.416 | -0.3909 | Yes |
| 65 | TIAL1 | TIA1 cytotoxic granule associated RNA binding protein like 1 [Source:HGNC Symbol;Acc:HGNC:11804] | 11049 | -0.438 | -0.3895 | Yes |
| 66 | FKBP6 | FKBP prolyl isomerase family member 6 (inactive) [Source:HGNC Symbol;Acc:HGNC:3722] | 11173 | -0.454 | -0.3855 | Yes |
| 67 | CNOT6 | CCR4-NOT transcription complex subunit 6 [Source:HGNC Symbol;Acc:HGNC:14099] | 11212 | -0.459 | -0.3747 | Yes |
| 68 | PIWIL2 | piwi like RNA-mediated gene silencing 2 [Source:HGNC Symbol;Acc:HGNC:17644] | 11219 | -0.460 | -0.3614 | Yes |
| 69 | AGO2 | argonaute RISC catalytic component 2 [Source:HGNC Symbol;Acc:HGNC:3263] | 11246 | -0.463 | -0.3495 | Yes |
| 70 | TDRD12 | tudor domain containing 12 [Source:HGNC Symbol;Acc:HGNC:25044] | 11283 | -0.469 | -0.3383 | Yes |
| 71 | SRRT | "serrate, RNA effector molecule [Source:HGNC Symbol;Acc:HGNC:24101]" | 11626 | -0.521 | -0.3494 | Yes |
| 72 | DDX17 | DEAD-box helicase 17 [Source:HGNC Symbol;Acc:HGNC:2740] | 11716 | -0.535 | -0.3403 | Yes |
| 73 | MIR22HG | MIR22 host gene [Source:HGNC Symbol;Acc:HGNC:28219] | 11785 | -0.546 | -0.3292 | Yes |
| 74 | TNRC6B | trinucleotide repeat containing adaptor 6B [Source:HGNC Symbol;Acc:HGNC:29190] | 11947 | -0.582 | -0.3243 | Yes |
| 75 | RIPK1 | receptor interacting serine/threonine kinase 1 [Source:HGNC Symbol;Acc:HGNC:10019] | 11953 | -0.584 | -0.3072 | Yes |
| 76 | MIR600HG | MIR600 host gene [Source:HGNC Symbol;Acc:HGNC:23642] | 12045 | -0.605 | -0.2962 | Yes |
| 77 | EGFR | epidermal growth factor receptor [Source:HGNC Symbol;Acc:HGNC:3236] | 12109 | -0.622 | -0.2825 | Yes |
| 78 | EIF4G1 | eukaryotic translation initiation factor 4 gamma 1 [Source:HGNC Symbol;Acc:HGNC:3296] | 12122 | -0.625 | -0.2647 | Yes |
| 79 | DICER1 | "dicer 1, ribonuclease III [Source:HGNC Symbol;Acc:HGNC:17098]" | 12215 | -0.650 | -0.2524 | Yes |
| 80 | DND1 | DND microRNA-mediated repression inhibitor 1 [Source:HGNC Symbol;Acc:HGNC:23799] | 12266 | -0.667 | -0.2363 | Yes |
| 81 | NEAT1 | nuclear paraspeckle assembly transcript 1 [Source:HGNC Symbol;Acc:HGNC:30815] | 12374 | -0.700 | -0.2237 | Yes |
| 82 | CELF1 | CUGBP Elav-like family member 1 [Source:HGNC Symbol;Acc:HGNC:2549] | 12391 | -0.704 | -0.2038 | Yes |
| 83 | IL6 | interleukin 6 [Source:HGNC Symbol;Acc:HGNC:6018] | 12460 | -0.730 | -0.1873 | Yes |
| 84 | PABPC1 | poly(A) binding protein cytoplasmic 1 [Source:HGNC Symbol;Acc:HGNC:8554] | 12562 | -0.778 | -0.1718 | Yes |
| 85 | ZC3H7B | zinc finger CCCH-type containing 7B [Source:HGNC Symbol;Acc:HGNC:30869] | 12639 | -0.815 | -0.1533 | Yes |
| 86 | CNOT9 | CCR4-NOT transcription complex subunit 9 [Source:HGNC Symbol;Acc:HGNC:10445] | 12668 | -0.839 | -0.1304 | Yes |
| 87 | AGO3 | argonaute RISC catalytic component 3 [Source:HGNC Symbol;Acc:HGNC:18421] | 12726 | -0.888 | -0.1082 | Yes |
| 88 | DDX3X | DEAD-box helicase 3 X-linked [Source:HGNC Symbol;Acc:HGNC:2745] | 12788 | -0.949 | -0.0845 | Yes |
| 89 | MEG3 | maternally expressed 3 [Source:HGNC Symbol;Acc:HGNC:14575] | 12825 | -1.016 | -0.0569 | Yes |
| 90 | DDX5 | DEAD-box helicase 5 [Source:HGNC Symbol;Acc:HGNC:2746] | 12834 | -1.038 | -0.0264 | Yes |
| 91 | ZFP36 | ZFP36 ring finger protein [Source:HGNC Symbol;Acc:HGNC:12862] | 12846 | -1.052 | 0.0042 | Yes |
Table: GSEA details [plain text format]

  

Fig 2: GOBP\_GENE\_SILENCING\_BY\_RNA      
 Blue-Pink O' Gram in the Space of the Analyzed GeneSet

  

Fig 3: GOBP\_GENE\_SILENCING\_BY\_RNA: Random ES distribution      
 Gene set null distribution of ES for **GOBP\_GENE\_SILENCING\_BY\_RNA**
